# Successful intravenous thrombolysis in an acute ischemic stroke patient with contraindicating values of the international standardized ratio and prothrombin time

**DOI:** 10.1007/s13760-020-01512-1

**Published:** 2020-10-09

**Authors:** Liming Cao, Xibao Tong, Hua Lu

**Affiliations:** 1 Department of Neurology, The Third Affiliated Hospital of Shenzhen University, 47 Friendship Road, Luohu District, Shenzhen, 518000 China; 2 Department of Internal Medicine, People’s Hospital of Xilin County, Baise, 533500 Guangxi China

Dear Editor,

Warfarin is used abundantly to treat atrial fibrillation and other diseases, because it reduces the relative risk of nonvalvular atrial fibrillation-related stroke by 64% and all-cause mortality by 26% [1]. However, warfarin cannot completely prevent cardiogenic stroke. Recombinant tissue plasminogen activator (rt-PA) is the most effective therapeutic drug for treating acute ischemic stroke (AIS) [2]. However, current guidelines [3, 4] contraindicate the use of intravenous thrombolysis (IVT) when the international standardized ratio (INR) is > 1.7 or the prothrombin time (PT) is > 15 s (level B evidence). IVT with rt-PA in warfarin-treated stroke patients beyond these guidelines is rare. We propose that warfarin-treated stroke patients with an INR > 1.7 or PT > 15 s should be individually assessed to determine if IVT is appropriate. Here, we report the case of an AIS patient with INR and PT values beyond the contraindications who underwent IVT and achieved good results.

A 53-year-old man was admitted to the emergency department for right limb weakness and speech impediment since 2 h. He had undergone artificial mechanical valve replacement in 2016 for aortic valve severe stenosis with valve regurgitation secondary to rheumatic heart valvular disease. After this, he was prescribed warfarin (3 mg/day). The last dose had been administered 10 h before the onset of symptoms.

The patient had no history of cardiovascular risk factors, such as smoking; drinking; hypertension; or diabetes, and infectious or genetic diseases. Physical examination showed blood pressure of 119/70 mmHg, somnolence, motor aphasia, right central facial paralysis, decreased muscle strength in right upper and lower limbs (4/5), positive right Babinski sign, and normal cardiac rhythm. His National Institutes of Health Stroke Scale (NIHSS) score was 6. Emergency computed tomography (CT) showed no obvious abnormalities (Fig. 1a, b). Blood coagulation test showed raised INR (2.18) and PT (25.63 s) (Table 1). Platelet count, blood electrolyte levels, random blood sugar levels, and electrocardiography findings were all normal.

**Fig. 1:**
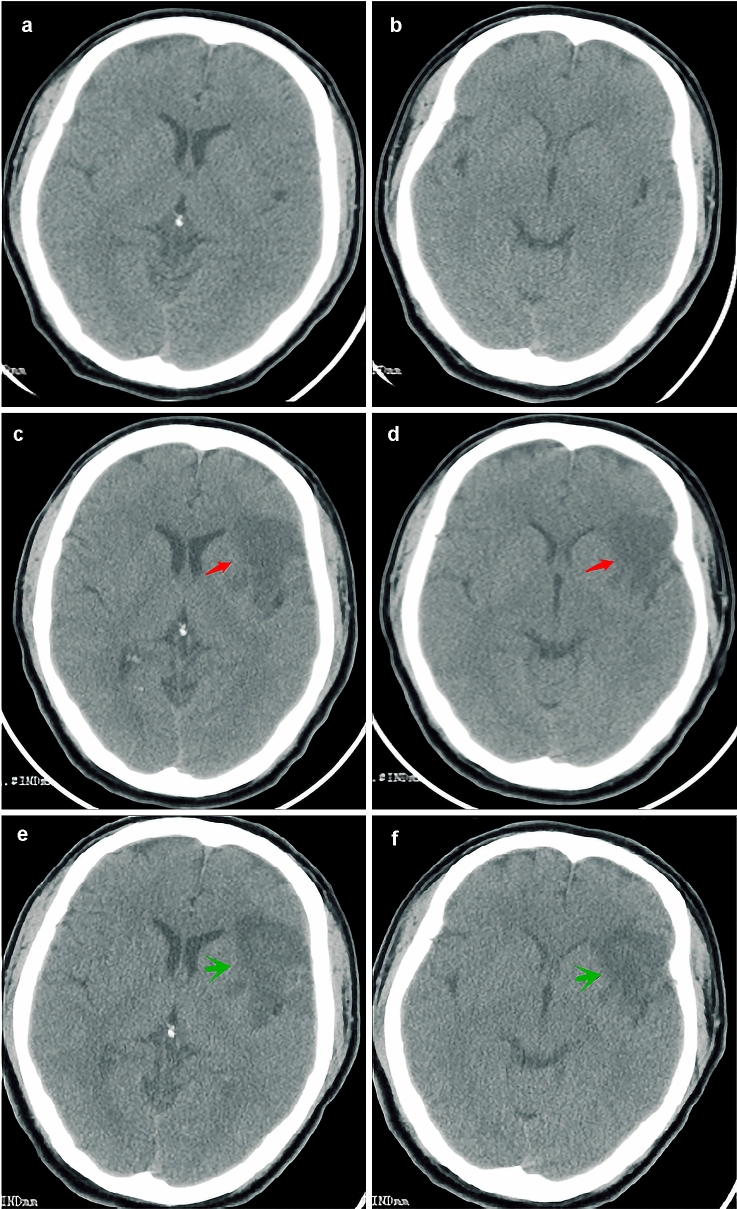
Emergency brain CT before intravenous thrombolysis showing no obvious abnormalities (**a**, **b**). Repeated brain CT showing an acute infarction in the frontotemporal area (arrow) and no hemorrhagic transformation at 35 h (**c**, **d**) and 1 week (**e**, **f**) after the onset. *CT* computed tomography

**Table 1:** Changes of blood coagulation function before and after intravenous thrombolysis

Coagulation function	11–20 21:43	11–21 0:30	11–21 11:49	11–24 11:05	11–26 10:03	11–28 09:28	Reference ranges
INR	2.18	2.13	1.79	1.15	1.42	2.08	0.8–1.25
PT, s	25.63	24.97	21.08	13.74	16.85	24.40	9.2–15.0
Fibrinogen level, g/L	1.76	1.52	1.48	2.10	2.00	2.03	2.0–4.0
APTT, s	33.73	36.28	35.7	29.15	30.56	34.66	21–37
TT, s	14.88	17.33	17.08	14.59	14.09	14.23	10–20

*APTT* activated partial thrombin time, *INR* international normalized ratio, *PT* prothrombin time, *TT* thrombin time

The patient was informed of the potential risks and benefits of IVT, and he provided consent for the procedure. IVT with rt-PA (0.8 mg/kg) was administered 3 h after the onset of symptoms, with an initial 0.9 mg/kg intravenous bolus injection, representing 10% of the total dosage. The remainder was administered intravenously over 1 h. We administered an intramuscular injection of vitamin K1 (10 mg) 30 min after IVT initiation.

His speech disorder greatly improved 2 h after IVT initiation (NIHSS = 5). His motor and speech functions gradually normalized within 9 h. The only residual symptom was mild facial paralysis (NIHSS = 1). The following laboratory parameters were elevated: white blood cell count, 16.34 × 10^9^/L; red blood cell count, 5.72 × 10^12^/L; neutrophil percentage, 87%; plasma D dimer level, 1.12 mg/L; and serum total bilirubin level, 19.5 μmol/L. Fasting blood glucose, serum creatinine, and serum cholesterol levels were normal. Carotid artery Doppler ultrasound and 24-h dynamic electrocardiography showed no obvious abnormalities. Echocardiography showed enhancement of the leaflets of the aortic valve (secondary to mechanical heart valve replacement), left-ventricular enlargement, and decreased left-ventricular systolic function. Repeated brain CT showed an acute infarct and no hemorrhagic transformation 35 h (Fig. 1c) and 1 week (Fig. 1d) after the onset of symptoms. The patient continued receiving his usual warfarin dose (3 mg/day) after 2 days of IVT. He felt no discomfort and was discharged on post-hospitalization day 9 (NIHSS = 0).

Cases reporting successful IVT with rt-PA in warfarin-treated stroke patients with INR > 1.7 and PT > 15 s are rare. Factors influencing successful IVT in our patient were young age [5]; lack of other underlying conditions such as trauma, digestive tract/urinary tract diseases, renal insufficiency, hypertension, and diabetes [6]; short time between onset of symptoms and IVT; and vitamin K administration to correct the elevated INR [7].

Mechanisms and processes of thrombus formation, IVT, and warfarin anticoagulation are interrelated (Fig. 2). A large cross-sectional study [8] found that the prognosis of patients with an INR > 1.7 was the same as that of the other patients. Two cases have successfully reported the use of IVT in patients presenting with AIS and an INR of 1.9 (the INR was not as high as our case, and PT and detailed changes of blood coagulation function were not mentioned) [9, 10]. Thus, IVT with rt-PA may not increase the risk of symptomatic intracranial hemorrhage (sICH) or poor prognosis in warfarin-treated stroke patients with an INR > 1.7.

**Fig. 2:**
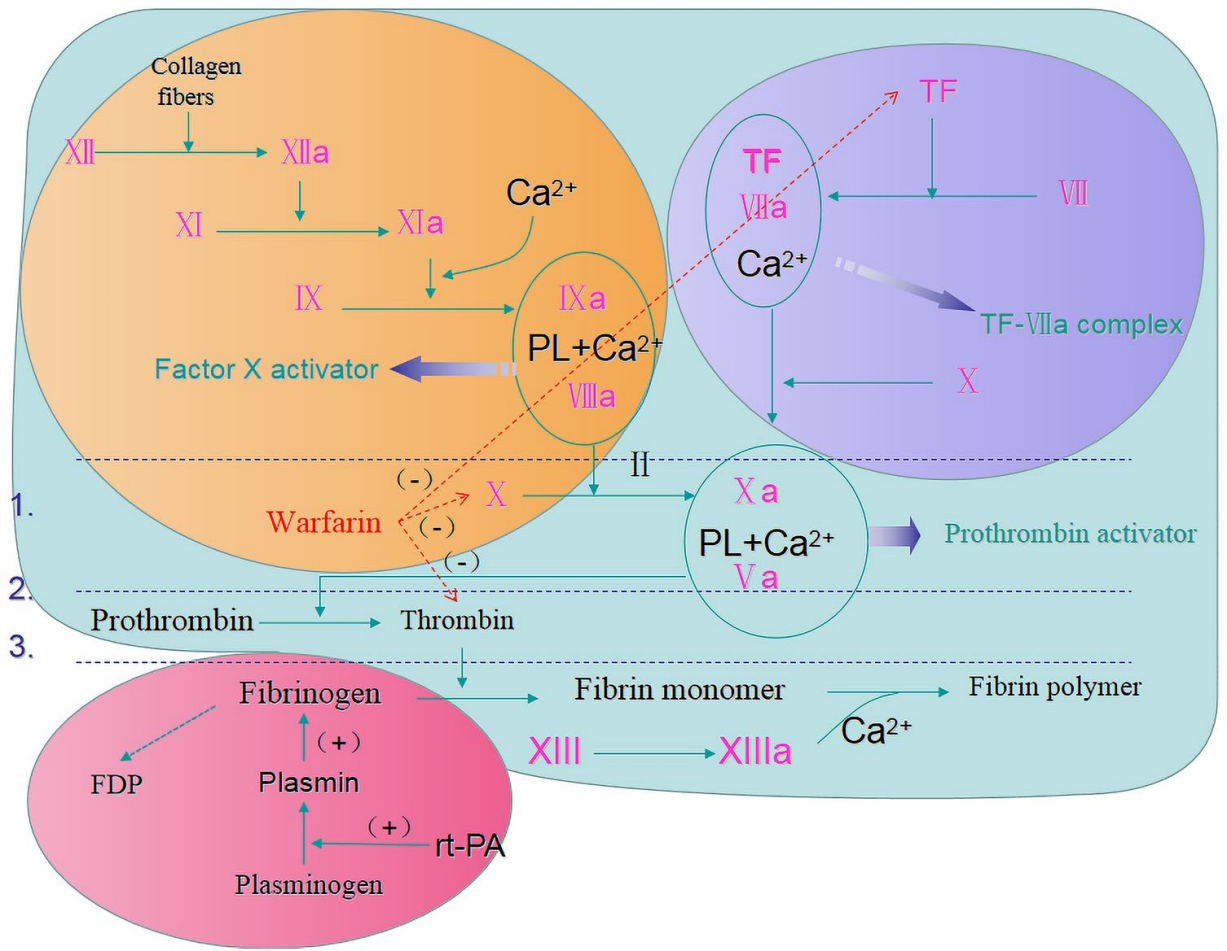
Mechanisms and processes of thrombus formation, intravenous thrombolysis, and warfarin anticoagulation. Warfarin can inhibit prothrombin, TF, and coagulation factors X and IX, prolong the international standardized ratio, and reduce thrombosis, although the risk of bleeding may be increased by intravascular thrombosis. The content of the purple, yellow, red, and light green background circles indicates the extrinsic route of blood coagulation, intrinsic coagulation pathway, mechanism of intravenous thrombolysis with rt-PA, and mechanism of thrombus formation, respectively. *FDP* fibrinogen degradation products, *rt-PA* recombinant tissue plasminogen activator, *TF* tissue factor, *(+)* means catalysis, *(−) *means inhibition

Due to insufficient data, the safety and effectiveness of IVT with rt-PA in patients with an INR > 1.7 or PT > 15 s cannot be confirmed. We propose that warfarin-treated stroke patients with an INR > 1.7 or PT > 15 s should be individually assessed for the appropriateness of IVT. This report provides important information about IVT use in AIS patients with INR and PT values beyond the levels recommended by current guidelines, points out the direction for further studies, and may ultimately benefit more warfarin-treated patients. The major limitation of the report is that it is based on a single case.

In summary, we hypothesize that IVT with rt-PA, administered within 3 h of onset of symptoms, does not significantly increase the risk of sICH in some warfarin-treated AIS patients with an INR > 1.7 or PT > 15 s. The level of evidence is not high, and a multicenter, randomized clinical trial is required to verify this hypothesis.

## Data Availability

All data related to this case report are contained within the manuscript.
